# Protective Effect of a Water-Soluble Carotenoid-Rich Extract of *Cordyceps militaris* against Light-Evoked Functional Vision Deterioration in Mice

**DOI:** 10.3390/nu14081675

**Published:** 2022-04-18

**Authors:** Bo-Yie Chen, Ho-Shin Huang, Kan-Jen Tsai, Jia-Lain Wu, Ya-Ting Chang, Ming-Chih Chang, Chun-Mei Lu, Shih-Liang Yang, Hsiang-Shang Huang

**Affiliations:** 1 Department of Optometry, Chung Shan Medical University, Taichung 40201, Taiwan; boychen@csmu.edu.tw (B.-Y.C.); gloriawu7@gmail.com (J.-L.W.); tinya0821@gmail.com (Y.-T.C.); 2 Department of Ophthalmology, Chung Shan Medical University Hospital, Taichung 40201, Taiwan; 3 Department of Research and Development, Bio-Ray Biotech. Co., Ltd., Pingtung 90846, Taiwan; adinol.huang@gmail.com (H.-S.H.); cmlu@bio-ray.com.tw (C.-M.L.); 4 Department of Medical Laboratory and Biotechnology, Chung Shan Medical University, Taichung 40201, Taiwan; kjt@csmu.edu.tw; 5 Department of Medical Education and Research, Jen-Ai Hospital, Taichung 41625, Taiwan; zon681123@gmail.com; 6 Department of Exercise Health Science, National Taiwan University of Sport, Taichung 40404, Taiwan; 7 Department of Chinese Medicine, Taichung Hospital, Ministry of Health and Welfare, Taichung 40343, Taiwan; 8 Department of Cardiology, Jen-Ai Hospital, Taichung 41625, Taiwan

**Keywords:** *Cordyceps militaris*, cordyxanthins, water-soluble carotenoids, retinal photodamage, photoreceptor, functional vision, visual contrast sensitivity function

## Abstract

Light-evoked retinal photodamage is considered an important factor contributing to functional vision deterioration and can even lead to light maculopathy or dry age-related macular degeneration. Loss of visual acuity (VA) and visual contrast sensitivity function (VCSF) are the major symptoms of retinal degenerative diseases. *Cordyceps militaris* is a carotenoid-rich Chinese medicinal fungus with antioxidant, anti-inflammatory, and immunomodulatory functions. *C. militaris* extract is a natural substance, and its bioactive constituents have been shown to confer health benefits, but their application in retinal tissue and functional vision protection in vivo remain incompletely understood. In the present study, we evaluated the influence of water-soluble, carotenoid-rich *C. militaris* extracts on the visual performance of light-damaged mouse retinas in vivo, using adult female CD-1^®^ (ICR) albino mice. We showed that oral administration of this *C. militaris* extract (10 mg/kg, twice daily) protected the neural retina tissue against light-evoked photoreceptor cell death, reduced Müller cell hypertrophic gliosis, and elevated GSH levels and promoted the recovery of VA- and VCSF-thresholds, especially for high spatial frequency-characterized vision. These results suggest that, probably because of its water-soluble carotenoids, *C. militaris* extract has the potential to prevent or treat light-induced visual dysfunction.

## 1. Introduction

Sunlight and artificial high-energy light are associated with the progression of light-evoked retinopathies (light maculopathy) [1,2]. Excessive oxidative photodegradation and toxic metabolites generated from dysregulation of the visual cycle are usually considered detrimental to the health of the retina [3,4,5]. Overproduction of light-evoked toxic byproducts, such as all-trans-retinal dimer and bis-retinoid N-retinyl-N-retinylidene ethanolamine (A2E), exacerbates dysfunction of the photoreceptor and retinal pigment epithelium [5]. Additionally, inducing phase II enzymes or increasing the GSH content in retinas might initially alleviate oxidative injury in retinas [6,7]. Several studies have indicated that carotenoid-related nutritional supplements, such as lutein [8,9,10], zeaxanthin [8,10], and crocin [11,12] may help lower the risk of light-evoked macular dysfunction. However, in the clinical setting, carotenoid-rich materials have been suggested for preventative or therapeutic interventions for retinal diseases, including light maculopathy, age-related macular degeneration, and myopic retinopathy [13,14,15,16,17].

*Cordyceps militaris* (L.) Fr. (*C. militaris*) is a carotenoid-rich medicinal fungus with a long history of widespread use in Chinese medicine, functional food substances, and health promotion supplements [18,19]. Its crucial pharmacological effects are anti-inflammatory, antioxidant, anti-aging, anti-tumor, anti-diabetic, anti-fatigue, neuroprotective, liver-protective, and reno-protective, including metabolic syndrome improvement and immunomodulation [19,20,21,22,23,24,25]. *C. militaris* pigments are a potential source of natural carotenoids, which are secondary metabolites in the fruiting body and mycelium. In particular, *C. militaris*-based water-soluble carotenoids, named cordyxanthins [26], with different characteristics from typical fat-soluble carotenoids, have been identified. Additionally, the active bio-ingredients cordycepin (3′-deoxyadenosine), adenosine derivatives, ergosterol analogs, polysaccharides, mannitol, and fibrinolytic enzymes are present in *C. militaris* extract [20,23,25]. Furthermore, the components and medical efficacy of *C. militaris* extract are based on the cultivation process, environmental conditions, and bio-industrial extraction methodology. This *C. militaris* extract enriched with water-soluble carotenoids has expanded the application field in health promotion. However, its application for retinal tissue and functional vision protection remains incompletely investigated.

Appropriate supplements of nutrients or antioxidants have been shown to delay the onset of or attenuate light-evoked photoreceptor injury and consequently improve the quality of functional vision [12,27,28]. In the current study, a water-soluble carotenoid-rich extract of *C. militaris* from the fruit body was investigated for its retinal protective effect against light-evoked retinal photodamage in a mouse model and to explore its potential in clinical applications for vision protection.

## 2. Materials and Methods

### 2.1. Animals

Adult female CD-1^®^ (ICR) albino mice (BioLASCO Taiwan Co., Ltd., Taipei, Taiwan) were used in our experiments. Mice aged 8–9 weeks, weighing 25–28 g, were housed in a standard controlled environment with a 12 h:12 h light–dark schedule and provided food and water in a pathogen-free environment. The experimental design and methods were approved by the Institutional Animal Care and Use Committee (IACUC2469) of Chung Shan Medical University in accordance with the Guide for the Care and Use of Laboratory Animals.

### 2.2. Material Sources and Qualitative Determination of Cordyxanthins

**Figure 1:**
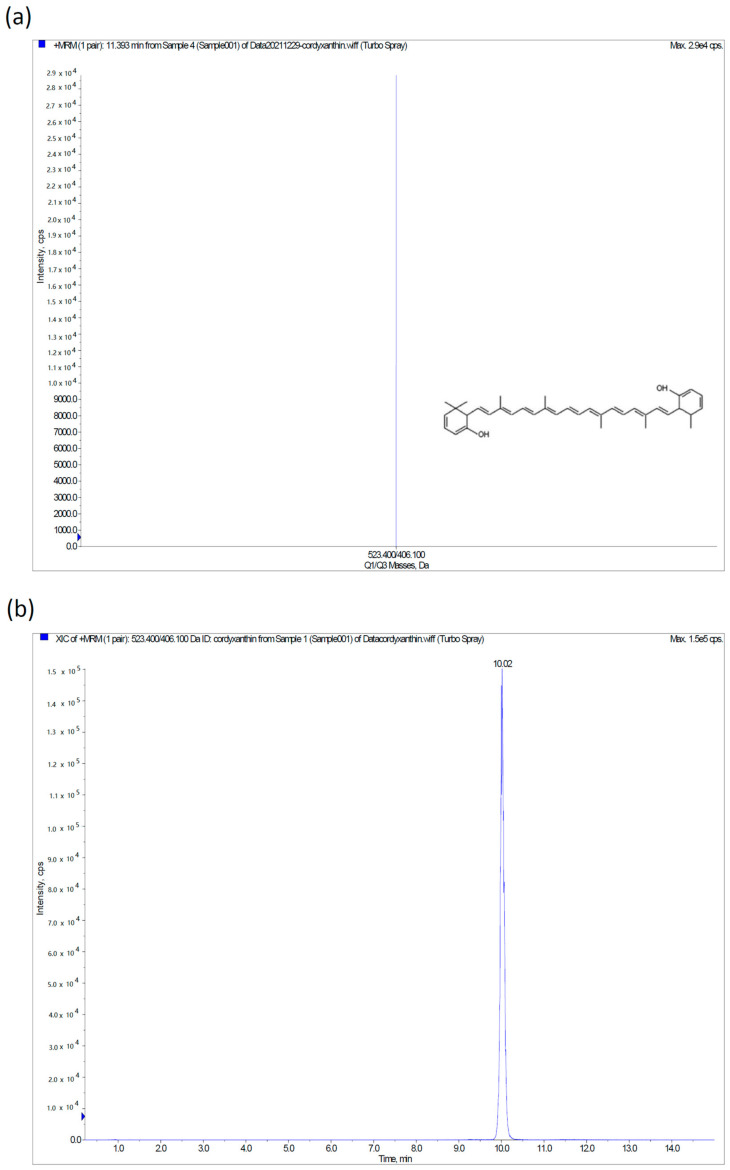
High-performance liquid chromatography-electrospray ionization mass spectrometry analysis of cordyxanthin, a water-soluble carotenoid, in *C. militaris* extract. (**a**) ESI source of the mass spectrometer; cordyxanthin-III showed good sensitivity in the positive ion mode; (**b**) MRM chromatographic profiles of the cordyxanthin from *C. militaris* extract.

A commercial marigold extract containing 20% lutein ester was used in this study. A water-soluble carotenoid-rich fraction (CrCM-1) isolated from *C. militaris* fruiting bodies using a Diaion HP column contained 0.8% cordycepin, 600 µg/g pigments, and cordyxanthin-rich substances (CrCM-1, Bio-Ray Biotech. Co., Ltd., Pingtung, Taiwan). The qualitative determination of cordyxanthin was performed and modified by high-performance liquid chromatography-electrospray ionization mass spectrometry (HPLC-ESI-MS, Applied Biosystems, Foster City, CA, USA) (Figure 1) with a multiple reaction monitoring methodology [26]. A Nexera XR-20A system (Shimadzu 8045, Kyoto, Japan) coupled with an API 4000 triple-quadrupole tandem mass spectrometer (Applied Biosystems, Foster City, CA, USA) was used for quantitative LC–MS/MS analysis, whereas the XBridge BEH C18 column (150 mm × 3.0 mm I.D, 2.5 μm; Waters, Ireland) was used for chromatographic separation (40 °C, 0.3 mL/min flow rate). The injection volume was 1 μL. The mobile phase consisted of 0.1% formic acid aqueous solution (solution A) and acetonitrile (solution B). The gradient elution program was performed as follows: solution A, 90–60% (0–7.5 min); 60–40% (7.5–10.8 min); 40–0% (10.8–19 min); 0–40% (19–21 min); 40–60% (21–25 min); and 50–90% (25–30 min). 

### 2.3. Experimental Design and Animal Groupings

**Figure 2:**
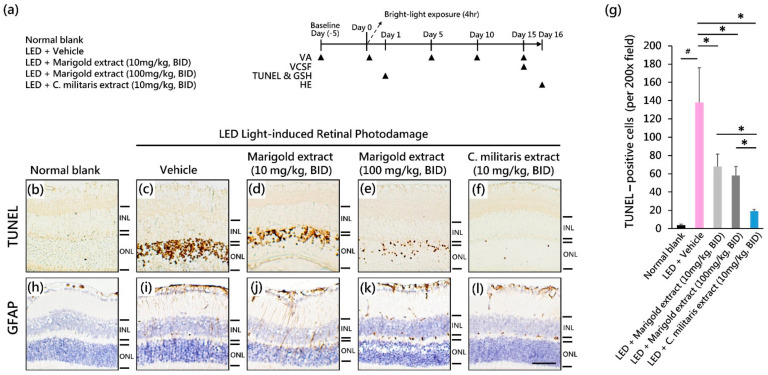
Protective effect of *C. militaris* extract on light-evoked retinal damage. (**a**) Timeline of experimental design; (**b**–**f**) The analysis of apoptotic cell death in retinas; (**g**) The density of apoptotic cells; (**h**–**l**) The expression of GFAP protein. Data are expressed as mean ± SE. Mann–Whitney U test was used to analyze data. ^#^: *p* < 0.05, *: *p* < 0.05. Scale bar: 35 μm. INL: the inner nuclear layer; ONL: the outer nuclear layer.

*C. militaris* extract (CrCM-1) was freshly prepared in a vehicle: distilled water containing 10% (*v*/*v*) propylene glycol 400. A lutein-rich marigold extract (20% lutein ester) was also prepared and emulsified in the same vehicle. Referring to our previous studies [12,27], a mouse model of light-emitting diode (LED)-evoked retinal damage was modified and induced at 13,000–17,000 lux of light intensity for 4 h (Figure 2a). Five experimental groups were set: (1) normal blank group (no light exposure) (*n* = 8); (2) LED + vehicle treatment group (*n* = 8); (3) LED + marigold extract-treated group (10 mg/kg, BID) (*n* = 8); (4) LED + marigold extract-treated group (100 mg/kg, BID) (*n* = 8); and (5) LED + *C. militaris* extract-treated group (10 mg/kg, BID) (*n* = 8). The vehicle, marigold extract, and *C. militaris* extract groups were administered by oral gavage twice daily starting from 5 days prior to bright light stimulation and persisting until day 16 before sacrifice.

For the retinal protection analysis (*n* = 4 per group), the mice were sacrificed on day 1 after light exposure (24 h after light exposure). The eyeballs were enucleated and perfused with a fixative solution to analyze apoptotic cell death in retinas by TUNEL (terminal deoxynucleotidyl transferase dUTP nick-end labeling) staining. In the study of functional vision (*n* = 4 per group), the timeline of visual acuity (VA) assessment was at day 5 (baseline), day 0 (after light exposure), day 5, day 10, and day 15 (Figure 2a). Visual contrast sensitivity function (VCSF) assessment was performed on day 15 (Figure 2a). All mice were adapted in dim conditions (50 ± 10 lux illuminance) for 0.5 h before the examination.

### 2.4. Determination of Thresholds of Visual Acuity (VA) and Visual Contrast Sensitivity Function (VCSF)

Referring to functional vision studies in mice [12,27,29,30], the reflexes of the optomotor response (OMR) could reveal the thresholds of VA and VCSF. The OMR approach was based on the detection of eliciting compensatory head movements when visual fields were exposed to the stimulus of moving striped grating patterns. When mice’s head movement behavior was no longer coordinated correctly with the movement of stimulus gratings, the threshold levels were determined by given spatial frequency stimuli [18,27,29,30]. The square waveform-characterized striped grating pattern, displaying equipment, conditions, and the recording methodology in this study were referred to in our previous research [12,27]. In the OMR-based VA test, the striped grating patterns were set on a series of spatial frequencies of 0.033, 0.055, 0.082, 0.164, 0.328, and 0.437 cycles per degree (cpd) with 100% contrast. In the OMR-based VCSF test, each grating stripe described above was set and divided into ten different contrast levels to determine the individual thresholds of spatial frequency stimulation. In the OMR-based VCSF test, these different contrast grating stripes were used to determine the individual thresholds of spatial frequency stimulation. These VCSF threshold values were obtained and then incorporated and graphed as an inverted U-shaped curve. The area under this curve (AUC) was calculated as a VCSF visibility index to represent the capacities of overall VCSF performance. In the VCSF test, if mice could rapidly and correctly respond to relatively lower-contrast stimuli, a higher VCSF curve and greater performance for functional vision would be obtained.

### 2.5. Histological Analyses and Immunohistochemistry

The eyeball was soaked in fixative solution for 24 h and embedded in paraffin. Tissue sections (6 μm thickness) were prepared in the sagittal plane for hematoxylin and eosin (H&E) staining or immunohistochemistry staining. The retinal microstructure was analyzed using an Olympus CX-22 microscope (Olympus Corp., Tokyo, Japan), a Motic Moticam 3 camera, and Motic Image Plus software (version 2.0; Motic, Xiamen, China). The nuclei count of the outer nuclear layer (ONL) in retinas was measured vertically at 200× magnification, and the average nuclei were calculated in the region of 0.4~1.0 mm to the superior and inferior central optic nerve head (ONH). For immunohistochemical staining, regular sodium citrate buffer was used for heat-induced antigen retrieval prior to incubation with the antibody. The anti-glial fibrillary acidic protein (GFAP) antibody (1/400, Cat. No. ab7260, Abcam, Cambridge, UK) was incubated with the retinal sections after antigen retrieval. Signals were detected using an immunohistochemistry kit (Super Sensitive™ Polymer-HRP IHC Detection System; BioGenex Laboratories, Inc., Fremont, CA, USA).

### 2.6. TUNEL (Terminal Deoxynucleotidyl Transferase dUTP Nick-End Labeling) Staining

The eyeball was fixed in 4% paraformaldehyde for 24 h at 4 °C and embedded in paraffin for sectioning (6 μm). The TdT-mediated dUTP nick-end labeling (TUNEL) was performed in the retinal sections according to the protocol of the TUNEL Assay Kit-HRP-DAB (ab206386, Abcam). TUNEL-positive nuclei were counted in the ONL (photoreceptor cell bodies) of retinas.

### 2.7. Glutathione Assay

The mouse retinal tissues were isolated and removed from the eyecup, homogenized, and centrifuged at 12,000× *g* for 10 min at 4 °C. The supernatants were subjected to GSH assay. Five micrograms of the total protein lysate were used. Retinal GSH in retinal tissues was determined by colorimetric assay using a commercial GSH assay kit (ADI-900-160, Enzo Life Sciences, Farmingdale, NY, USA).

### 2.8. Statistical Analysis

In the quantitative test, data are presented as mean ± standard error (SE), and statistical analysis was performed using SPSS v. 22 software (IBM Corp., Armonk, NY, USA). Differences between groups were analyzed using the Kruskal–Wallis test and Mann–Whitney U-test. Significant differences were shown by *p* < 0.05, and *p* < 0.01.

## 3. Results

### 3.1. Qualitative Determination of Cordyxanthin in C. militaris Extract

High-performance liquid chromatography-electrospray ionization mass spectrometry (HPLC-ESI-MS, Applied Biosystems, Foster City, CA, USA) with multiple reaction monitoring methodology was used for the qualitative determination of cordyxanthin. An RP-C18 column (XBridge BEH 2.5 μm, 150 mm × 3 mm, Waters, Ireland) was selected for this analysis. With the ESI source of the mass spectrometer, cordyxanthin showed good sensitivity in the positive ion mode (Figure 1a). The precursor-to-product ion transitions were m/z 523.4/406.1, which were comparable to those reported in the literature [26]; thus, it might be speculated to be cordyxanthin-III. The MRM chromatographic profile of cordyxanthin is shown in Figure 1b. The *C. militaris* extract used in this study was confirmed to be rich in cordyxanthin, a water-soluble carotenoid.

### 3.2. Suppression of Light-Evoked Apoptotic Cell Death with C. militaris Extract

To elucidate the oral efficacy of the *C. militaris* extract on light-evoked apoptotic cell death of photoreceptors in vivo, a TUNEL assay (Figure 2b–f) was performed on the retinas. In age-matched normal retinas that received no light exposure, the number of TUNEL-positive cells was rare to negligible (Figure 2b). In contrast, light-evoked vehicle-treated mice showed substantial cell apoptotic death in the photoreceptor cell body-rich ONL (Figure 2c). The number of light-evoked TUNEL-positive cells was significantly attenuated by marigold extract (100 mg/kg, BID) (Figure 2e) or *C. militaris* extract (10 mg/kg, BID) (Figure 2f). The inhibition of apoptosis was significantly increased by the application of *C. militaris* extract (10 mg/kg, BID) compared to vehicle-treated, marigold extract-treated (10 mg/kg, BID), and marigold extract-treated (100 mg/kg, BID) groups (*p* < 0.05, Figure 2g). Treatment with *C. militaris* extract resulted in a significant protective effect against light-induced photoreceptor damage.

### 3.3. Suppression of Light-Evoked Müller Cell Gliosis with C. militaris Extract

To evaluate the effect of *C. militaris* extract on light-evoked pathological changes in retinal Müller cells, GFAP staining was performed 24 h after light exposure. Light exposure to vehicle-treated mice led to Müller cell gliosis with GFAP expression (Figure 2i) as compared to normal retinas receiving no light exposure (Figure 2h). The efficacy of suppression of light-evoked Müller cell gliosis was substantial in mice that were marigold extract-treated (100 mg/kg, BID) (Figure 2k) and *C. militaris* extract-treated (10 mg/kg, BID) (Figure 2l), respectively, as compared to marigold extract-treated (10 mg/kg, BID) mice (Figure 2j). *C. militaris* extract treatment resulted in significant protective efficacy against light-evoked Müller cell gliosis.

### 3.4. C. militaris Extract Rapidly Restores Visual Acuity against Light-Evoked Deterioration

**Figure 3:**
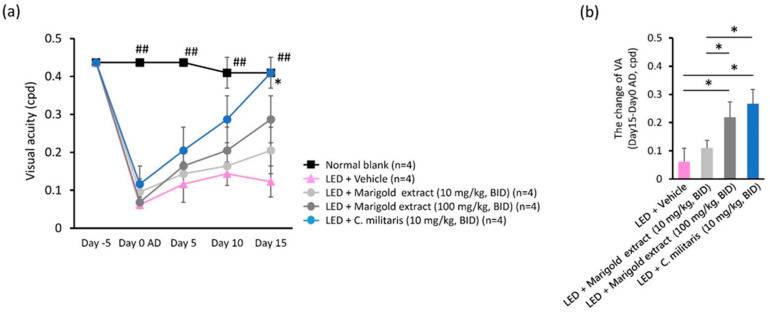
Effects on visual acuity (VA) of *C. militaris* extract treatment. (**a**) Changes in VA threshold after light-evoked retinal photodamage; (**b**) restorative efficacy of VA threshold of water-soluble carotenoid-rich, *C. militaris* extract-treated and lutein-rich, marigold extract-treated mice. Data are expressed as mean ± SE. A Mann–Whitney U test was performed; ^##^: *p* < 0.01, *: *p* < 0.05.

To investigate the effect of *C. militaris* extract on light-evoked vision deterioration, OMR-based visual acuity was assessed at five evaluation points over 16 days, i.e., day 5 before light exposure and day 0, day 5, and day 15 after light exposure (Figure 3a). Light exposure to vehicle-treated mice led to a significant reduction in the threshold of VA compared to the normal retina receiving no light exposure (Figure 3a). Oral administration of marigold extract (10 mg/kg, BID and 100 mg/kg, BID) and *C. militaris* extract (10 mg/kg, BID) enabled light-evoked mice to recover from the reduction in the VA threshold after light exposure (Figure 3a). The recovery value of the VA threshold was 0.109 ± 0.027 cpd in marigold extract-treated mice (10 mg/kg, BID), 0.219 ± 0.055 cpd in marigold extract-treated mice (100 mg/kg, BID), and 0.266 ± 0.051 cpd in *C. militaris* extract-treated mice (10 mg/kg, BID) (Figure 3b). The recovery efficacy of the VA threshold was significantly better with *C. militaris* extract (10 mg/kg, BID) than with marigold extract (10 mg/kg, BID) (*p* < 0.05, Figure 3b). Importantly, these results indicate that the *C. militaris* extract exerted protective or restorative properties in response to light-evoked visual acuity deterioration.

### 3.5. C. militaris Extract Preserves High Spatial Frequency-Based Vision against Bright Light

**Figure 4:**
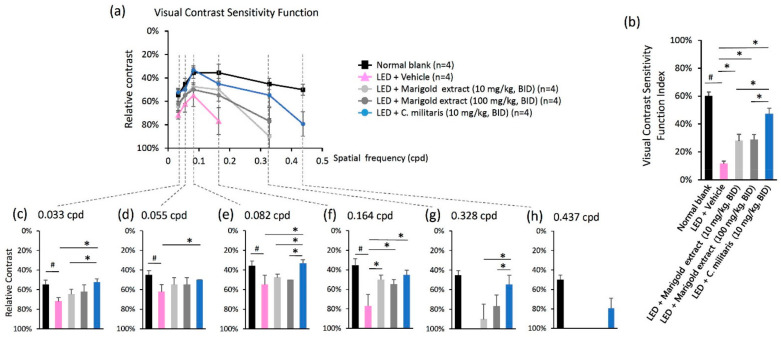
Effects on visual contrast sensitivity function (VCSF) of *C. militaris* extract treatment. (**a**) The inverted U-shaped diagram of the VCSF curve; (**b**) The VCSF visibility index; (**c**) The individual threshold with details represented in 0.033 cpd, (**d**) 0.055 cpd, (**e**) 0.082 cpd, (**f**) 0.164 cpd, (**g**) 0.328 cpd, and (**h**) 0.437 cpd. Data are expressed as mean ± SE. A Mann-Whitney U test was performed. ^#^: *p* < 0.05, *: *p* < 0.05.

The inverted U-shaped VCSF curve represents the capacity for visual performance (Figure 4a). The area under this curve (AUC) was calculated as the VCSF visibility index (Figure 4b), which increased with amplification of the VCSF performance. Light exposure of vehicle-treated mice led to a significantly reduced VCSF curve compared to the normal retina receiving no light exposure (Figure 4a). Noticeably, higher VCSF curves were shown in *C. militaris* extract-treated mice (10 mg/kg, BID) (Figure 4a) than those of the marigold extract-treated mice (10 mg/kg, BID and 100 mg/kg, BID) (Figure 4a). The VCSF visibility index was significantly increased in *C. militaris* extract-treated mice (10 mg/kg, BID) at 47.22 ± 4.11%, marigold extract-treated mice (10 mg/kg, BID) at 28.05% ± 4.59%, and marigold extract-treated mice (100 mg/kg, BID) at 28.96% ± 3.51% as compared to vehicle-treated mice at 11.51 ± 1.87% (Figure 4b). The VCSF visibility index of the normal blank was shown at 60.07 ± 2.86% (Figure 4b).

In particular, high spatial frequency-based VCSF was protected in *C. militaris* extract-treated mice (10 mg/kg, BID), especially presented at 79.33 ± 10.34% at 0.437 cpd, while it was not detectable in the vehicle- and marigold extract-treated mice (10 mg/kg, BID and 100 mg/kg, BID) (Figure 4h). By comparison, *C. militaris* extract- and marigold extract-treated mice also showed improved thresholds at a high spatial frequency of 0.328 cpd (Figure 4g), while it presented at 54.75% ± 9.50% in the *C. militaris* extract-treated mice (10 mg/kg, BID), 89.90 ± 15.14% in the marigold extract-treated mice (10 mg/kg, BID), and 76.92 ± 11.54% in the marigold extract-treated mice (100 mg/kg, BID). Similar results were observed in the middle- to low-level spatial frequency analysis of 0.164 cpd (Figure 4f), 0.082 cpd (Figure 4e), 0.055 cpd (Figure 4d), and 0.033 cpd (Figure 4c). However, based on this model, the power of VCSF protective efficacy of water-soluble, carotenoid-rich *C. militaris* extract was substantially greater than that of lutein-rich marigold extract, especially the high spatial frequency-based VCSF.

### 3.6. C. militaris Extract Suppression of Light-Induced ONL Degeneration

**Figure 5:**
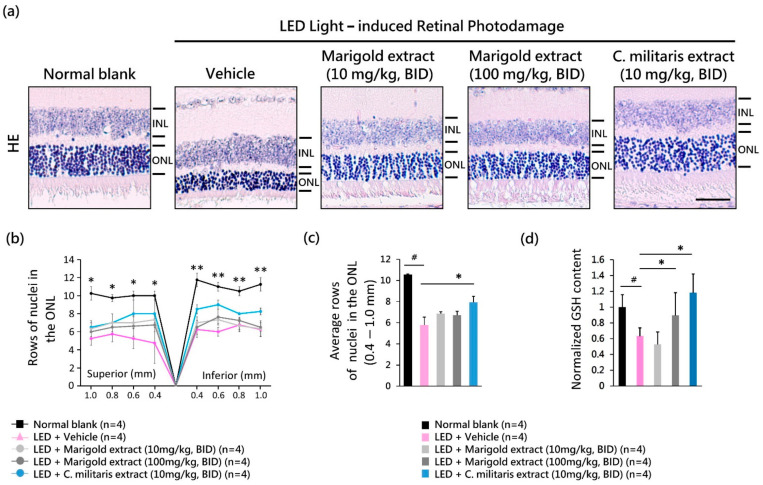
Antioxidant protective effect of *C. militaris* extract. (**a**) Hematoxylin and eosin staining representing the layers of ONL nuclei; (**b**) change of ONL nuclei within 0.4~1.0 mm superior and inferior to the optic nerve; (**c**) average ONL nuclei; (**d**) normalized GSH level. Data are expressed as mean ± SE. A Mann-Whitney U test was performed. ^#^: *p* < 0.05, *: *p* < 0.05, **: *p* < 0.01. Scale bar: 35 μm. INL: the inner nuclear layer; ONL: the outer nuclear layer.

To evaluate the protective effect of *C. militaris* extract on light-evoked histological damage to the retina, the layers of nuclei in ONL were analyzed on day 16 (Figure 5a). Light exposure of vehicle-treated mice led to a significant reduction in ONL nuclei compared to normal blank mice that received no light exposure (Figure 5a, and Figure 5b). In contrast, the loss of ONL nuclei induced by light exposure was significantly suppressed by *C. militaris* extract (10 mg/kg, BID) application (Figure 5b, and *p* < 0.05, Figure 5c). The protective effects of water-soluble, carotenoid-rich *C. militaris* extract (10 mg/kg, BID) were better than those of lutein-rich marigold extract (Figure 5b,c).

### 3.7. C. militaris Extract Present in Retinas Contributes to Antioxidant Capacities

To clarify whether the in vivo GSH content involved in *C. militaris* extract treatment contributes to retinal protection, the GSH level was measured in the retina tissue on day one. The light-evoked suppression of GSH levels in the retinas was reversed by treatment with *C. militaris* (10 mg/kg, BID) (*p* < 0.05, Figure 5d). Overall, these results indicate that the protective effect of *C. militaris* extract might be due to the water-soluble carotenoid pigments or its active ingredients penetrating the retina–blood barrier and persisting within the neural-retina tissue to modulate the detoxification pathway.

## 4. Discussion

Water-soluble, carotenoid-rich *C. militaris* is a highly valued edible and medicinal fungus [21,22,23,24]. The present study revealed the protective effect of *C. militaris* extracts on light-evoked retinal damage and the underlying capacities for promoting recovery of functional vision in mice. The light-induced deterioration of functional vision measured by OMR-based visual acuity (VA) and visual contrast sensitivity function (VCSF) were significantly suppressed by *C. militaris* treatment. In addition, the present study also indicated that the application of *C. militaris* extracts ameliorated several parameters in retinas, including light-evoked apoptotic cell death, Müller cell gliosis, ONL anatomical structure thinning, and GSH changes.

Reduced GSH content is a key factor in the pathogenesis of retinal damage [31,32]. In the present study, we showed that *C. militaris* extract treatment reversed the reduction in GSH levels resulting from the depletion of light exposure. In addition to protection of retinal tissue by *C. militaris* extracts, their contribution to vision performance has been confirmed in this mouse model. The present study revealed, for the first time, that the effect of *C. militaris* extracts in vivo on functional vision promotion is significant. In particular, our results revealed that the protective efficacy of *C. militaris* extracts (10 mg/kg, BID) on VCSF in mice was substantially higher than that of lutein-rich marigold extract (20% lutein esters) (10 mg/kg, BID and 100 mg/kg, BID), especially for high spatial frequency-characterized VCSF. By comparison, in humans, high spatial frequency sensitivity-characterized high-acuity vision is predominantly initiated by cone-rich macular vision, whereas temporal sensitivity is higher in peripheral vision than in central vision [33]. However, the cellular effects of *C. militaris* extract on cone- or rod-photoreceptor responses require further study.

The qualitative and quantitative detection of water-soluble carotenoid pigments of *C. militaris* extracts, the cordyxanthins, in materials or in biological samples has always been a challenge because of the lack of a natural chemical standard [26]. Owing to the limitations of methods for their quantitative analysis, the bioavailability of cordyxanthins remains unclear. Nonetheless, based on the water-soluble, carotenoid-enriched characteristics of the fruiting body or cultured mycelia, *C. militaris* is a bio-functional food source in a wide range of health supplements, and is incorporated or expanded in several health promotion applications in the food industry [20,21,22,23,24,25]. However, the pharmacological dosage is based on the composition of the extracts, including the content and proportion of cordycepin, adenosine derivatives, polysaccharides, ergosterol analogs, mannitol, peptides, and carotenoid pigments [20,22]. On the other hand, *C. militaris* has been commercially cultivated, and the carotenoid-enriched content is usually a superior-quality symbol of the production or industrial strategy [34,35,36,37,38].

Moreover, in the human retina, the macular pigments are composed of xanthophylls, but an insufficiency of carotenoids, including lutein, zeaxanthin, and meso-zeaxanthin, in the diet can alter macular pigment optical density (MPOD) and affect retinal health and functional vision [14,16,17,39]. Clinically, oral supplements of lutein- and zeaxanthin-based carotenoids are usually suggested, although it might be via scavenging free radicals to protect the macula from damage as well as recovering MPOD to improve vision [16,17]. The fruiting bodies of *C. militaris* contain fat-soluble β-carotene, lycopene, lutein, ze-axanthin, and water-soluble carotenoids [34,35,36,37,38]. The enriched β-carotene content in the fruiting bodies of *C. militaris* might also provide a source of vitamin A, which is crucial for the vision and visual cycle. In this study, we found that oral nutritional prophylaxis with *C. militaris* extracts in vivo in mice provided retinal tissue protection, while consequently resulting in recovery capacities for functional vision to alleviate light-evoked retinal damage. However, it is not yet known whether the pigments of *C. militaris*, water-soluble carotenoids or cordyxanthins, could penetrate and persist in the retina, contributing to enhancing antioxidant activity, directly scavenging reactive oxygen species, or promoting functional vision. Moreover, future studies are needed to reveal the underlying pharmacokinetics, toxicology, and mechanisms of action of *C. militaris* extract and cordyxanthin in retinas.

In summary, our results demonstrate the beneficial effects of water-soluble, carotenoid-rich *C. militaris* extract on retinas in a mouse model of light-induced damage. The protection afforded by *C. militaris* extract is primarily reflected in a high spatial frequency-characterized visual performance. In conclusion, *C. militaris* extract could be a potential candidate nutritional material for eye healthcare, especially for high-acuity vision healthcare.

## Data Availability

Not applicable.
